# Interprofessional assessment of medical students’ competences with an instrument suitable for physicians and nurses

**DOI:** 10.1186/s12909-019-1473-6

**Published:** 2019-02-06

**Authors:** Sarah Prediger, Sophie Fürstenberg, Pascal O. Berberat, Martina Kadmon, Sigrid Harendza

**Affiliations:** 1 Department of Internal Medicine, University Medical Center Hamburg-Eppendorf, Martinistr. 52, D-20246 Hamburg, Germany; 2 TUM Medical Education Center, School of Medicine, Technical University of Munich, München, Germany; 3 Faculty of Medicine, III. Medizinische Klinik, University of Augsburg, Deanery, Augsburg, Germany

**Keywords:** Competences, Competence-based assessment, Interprofessional differences, Rater-based assessment, Rater cognition, Resident, Undergraduate medical education

## Abstract

**Background:**

Physicians need a set of specific competences to perform well in interprofessional teams in their first year of residency. These competences should be achieved with graduation from medical school. Assessments during undergraduate medical studies are mostly rated by supervisors only. The aim of our study was to compare the rating of core facets of competence of medical students late in their undergraduate training as well as the rating confidence between three different groups of assessors (supervisors, residents, and nurses) in an assessment simulating the first day of residency.

**Methods:**

Sixty-seven advanced medical students from three different medical schools (Hamburg, Oldenburg and Munich) participated in a 360-degree assessment simulating the first working day of a resident. Each participant was rated by three assessors – a supervisor, a resident and a nurse – in seven facets of competence relevant for the first year of residency: (1) responsibility, (2) teamwork and collegiality, (3) knowing and maintaining own personal bounds and possibilities, (4) structure, work planning and priorities, (5) coping with mistakes, (6) scientifically and empirically grounded method of working, and (7) verbal communication with colleagues and supervisors. Means of all assessed competences and confidences of judgement of the three rating groups were compared. Additionally, correlations between assessed competences and confidence of judgement within each group of raters were computed.

**Results:**

All rating groups showed consistent assessment decisions (Cronbach’s *α*: supervisors = .90, residents = .80, nurses = .78). Nurses assessed the participants significantly higher in all competences compared to supervisors and residents (all *p* ≤ .05) with moderate and high effect sizes (*d* = .667–1.068). While supervisors’ and residents’ ratings were highest for “teamwork and collegiality”, participants received the highest rating by nurses for “responsibility”. Competences assessed by nurses were strongly positively correlated with their confidence of judgment while supervisors’ assessments correlated only moderately with their confidence of judgment in two competences.

**Conclusions:**

Different professional perspectives provide differentiated competence ratings for medical students in the role of a beginning resident. Rating confidence should be enhanced by empirically derived behavior checklists with anchors, which need to be included in rater training to decrease raters’ subjectivity.

**Electronic supplementary material:**

The online version of this article (10.1186/s12909-019-1473-6) contains supplementary material, which is available to authorized users.

## Background

Medicine has a long history of assessing performance by relying on the observation and judgement of teachers and experts, mostly because many aspects of clinical training cannot be assessed with knowledge tests but require demonstration and observation of skills or behaviour [1]. In clinical encounters, three variables are involved in the assessments: the learner, the patients, and the examiner. Ideally, patients and examiner should be held constant across all the learners to be assessed [2]. Common conceptualizations of observed rater cognition developed from the literature describe an underlying three-phase framework of rating: (1) identifying relevant information about the candidate (observation of performance), (2) giving meaning to the collected information (processing), and (3) forming an over-all judgement of the performance and rating (integration and rating) [3]. Despite such concepts, rater-based assessment is – like other social interaction – often based on an assessor’s first impression of a candidate [1], which can be overcome, though, when the performance of a candidate changes [4]. The rating context can have an impact on the willingness of raters to adjust a first impression-based rating [5]. For example, a negative cue would carry a greater weight, if the examiner fears to pass an examinee who should not pass [4], and could lead to an unwillingness to change a negative first impression.

Additionally, assessors’ reasoning in judgement is guided by their mental models of performance assessment, but these models are not necessarily universally shared [6]. When competences are assessed globally, rating can be strongly influenced by subjective impressions [7]. Therefore, different competences should be assessed separately as facets of competence [8] to give raters the opportunity to focus on each competence individually. This is eased by using conceptual rating models, which aim to assess exclusively relevant features based on theoretical and empirical grounds [9]. In general, a competence-based assessment can only be as good as the amount of work that was invested in the operationalization of measuring competence [10].

First attempts to operationalize observing and assessing clinical skills of undergraduate medical students in simulated clinical situations were objective structured clinical examinations (OSCE) [11]. With this method of examination, the students are assessed at a number of different stations by more than one rater, mostly physicians, which leads to more reliable and valid scoring outcomes by using standard setting criteria (e.g. station pass rates) and scoring checklists [12]. Better reliability was observed with a higher number of stations and a higher number of examiners per station, while interpersonal skills were evaluated less reliably across stations [13]. Peers have also been shown to rate other medical students’ skills in an OSCE reliably [14]. While OSCEs usually do not involve real patients and mostly assess skills, mini-clinical evaluations (Mini-CEX) [15] and direct observed procedural skills assessment (DOPS) [16] are used to assess skills, attitudes, and competences of students in the real clinical setting with real patients and to give feedback on the students’ performance. While OSCEs are mostly used for summative assessments, arguments and findings point towards using Mini-CEX and DOPS in a formative way with feedback for learning [17, 18]. However, satisfactory reliabilities for such workplace-based assessments can be reached and depend on the number of assessors observing at least two encounters or procedures each [19].

Besides changes in assessment tools, development towards competence-based education has occurred, which subsequently leads to a more prominent focus on direct observation for assessing learners [20]. The CanMEDS model, developed by the Royal College of Physicians and Surgeons of Canada, is a widely accepted framework of physicians’ competences for postgraduate medical education [21]. To ease the transition from undergraduate to postgraduate medical training, catalogues of competence-based learning objectives have also been developed for undergraduate medical studies [22, 23]. Certain core competences, e.g. empathy, interprofessional communication, and others with particular relevance for a beginning resident have been identified [8, 24], but the assessment of specific non-medical competences remains difficult [25, 26]. However, for competence-based education a robust and multifaceted assessment system is required [20].

For this study, we extended and re-designed an assessment of competences relevant for recently graduated physicians in the role of a beginning resident [27], which had been developed earlier [8]. Within this assessment, the student participants were rated in their role as residents by supervisors, residents, and nurses, with a competence-rating tool operationalized for seven different facet of competence. The first aim of our study was to compare the three different assessor groups with respect to their rating scores for the different facets of competence to identify, whether the assessment instrument is suitable for the three rater groups. Our second aim was to analyse the association of assessment scores in comparison with the confidence of rating within each rater group, to provide further insight into rater cognition.

## Methods

**Fig. 1:**
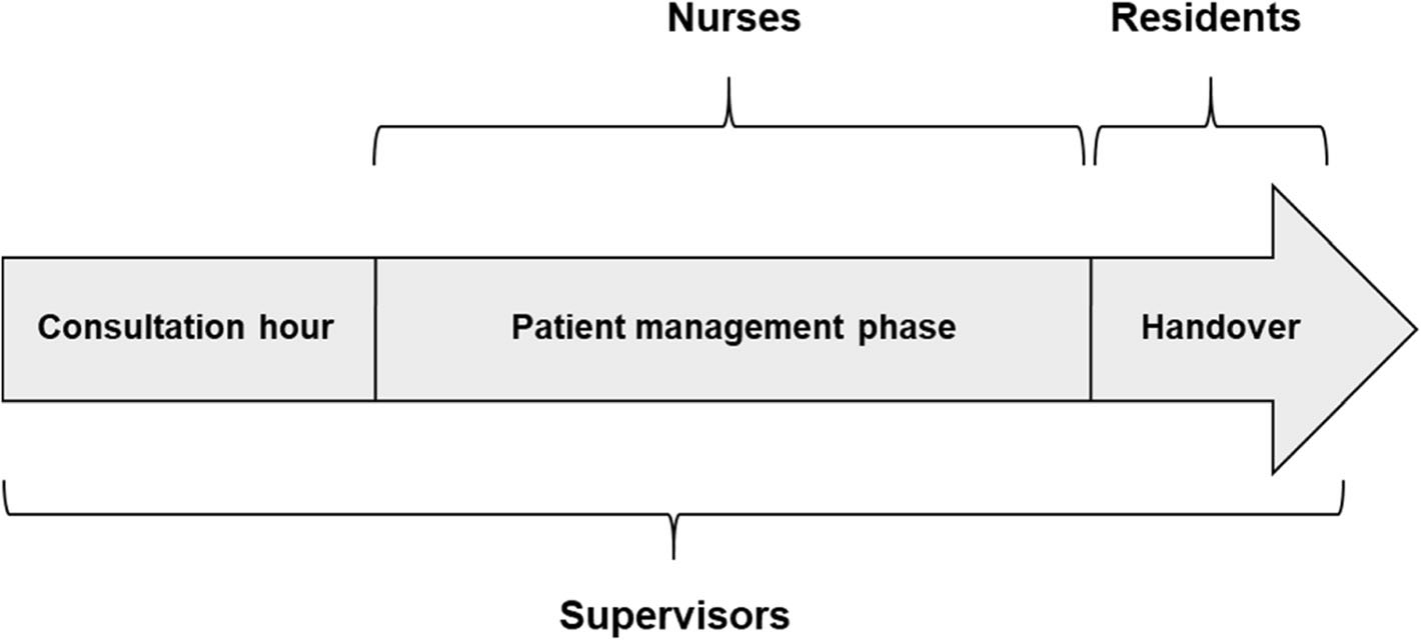
Phases of the assessment and observation by raters

Based on a ranking study of competences needed for the first year of residency [24], a 360-degree assessment of advanced medical students’ competences was developed [27]. The assessment simulates the first working day of a resident with three phases: a consultation hour with five simulated patients, followed by a patient management phase (2.5 h) for these five patients and interactions with nurses, and a handover phase of 30 min to a real resident (Fig. 1).The supervisors welcome their individual student in the role of a beginning resident before the consultation hour and meet them again in a face-to-face situation during the patient management phase for a short briefing about progress and questions. Additionally, the participants can call their supervisors on their cellular phone during all phases of the assessment to ask questions or to report certain aspects. The supervisors are also present during the handover, but do not interact with the participants or resident. The nurses collaborate with the students during the patient management phase in four interactions, where e.g. a patient deteriorates and the nurses ask the students in their role as residents for instructions. The participants can also interact with the nurses on their cellular phone, if they wish to. The residents only interact with the participants during the handover.

The participants were assessed by one supervisor, one resident, and one nurse in the following seven facets of competence: (1) responsibility, (2) teamwork and collegiality, (3) knowing and maintaining own personal bounds and possibilities, (4) structure, work planning and priorities, (5) coping with mistakes, (6) scientifically and empirically grounded method of working, and (7) verbal communication with colleagues and supervisors. Each facet of competence includes a definition that could be used as guideline by the assessors for observing performance. All assessors used the same instrument during the simulation to assess the students in these seven facets of competence (5-point-scales: 1 “insufficient” to 5 “very good”). It was also possible to skip the judgement as “no judgement possible”, if an assessor felt that a certain facet could not be observed in a participant. Furthermore, all raters had to assess the confidence of their judgement for every facet of competence (5-point-scale: 1 “uncertain” to 5 “certain”). A sample of the rating form can be found in Additional file 1. Nurses had to complete their ratings at the end of the patient management phase, supervisors and residents had to complete their ratings at the end of the handover phase. All raters were trained a few weeks before the assessment to learn how to use the rating instrument. They rated two videos of an excellent and a mediocre student taking a simulated patient’s history and discussed their judgements to build shared mental models [6] for each facet of competence.

The assessment took place during three days in July 2017 at the University Medical Center Hamburg-Eppendorf (UKE). 70 students from three universities, Hamburg, Oldenburg and Munich, with different curricula completed the study. Three of them had not reached year five of their undergraduate studies yet and were excluded from the statistical analysis. 54.3% of the 67 included undergraduate medical students were female. Their mean age were 25.9 ± 2.2 years. 41 students were in their final practice year, 26 had not started their final year yet. They were assessed by seven supervisors (six male and one female, two from Hamburg, two from Oldenburg, and three from Munich), five residents (two male and three female, all from Hamburg), and three nurses (one male and two female, all from Hamburg). The participants were assigned to the raters by university affiliation (e.g. students from Hamburg were assessed by supervisors from Munich or Oldenburg).

For statistical analysis, means and standard deviations were calculated for all assessed facets of competence and confidences of judgement for each of the three rating groups (supervisor, residents, and nurses) on SPSS Statistics 23. Cronbach’s *α* was calculated for reliability. To analyse differences between the rating groups we conducted an analysis of variance (ANOVA) and a Bonferroni post-hoc test as well as effect sizes (Cohen’s *d*). Additionally, we adjusted nurses’ facets of competence scores by reducing them according to the delta between supervisors’ mean score and nurses’ mean score. Pearson’s correlation coefficient (*r*) between the assessed facets of competence and confidence of judgement within each group of raters was computed, too.

## Results

The internal consistency of the ratings was .90 for supervisors, .80 for residents and .78 for nurses. Nurses assessed the participants significantly higher in all facets of competence compared to supervisors and residents (all *p* ≤ .05) with moderate and high effect sizes (*d* = .667–1.068), while ratings of both groups of physicians showed no significant differences in any of the facet of competence (Table 1). The comparison of adjusted nurses’ scores and supervisors’ scores in an individual ranking per facet of competence showed an average agreement of 42.8% of both rating groups, with higher agreements for participants, who were assessed “good” or “very good” (data not shown).

**Table 1:** Assessments of the students’ competences by different rating groups

Competences	Supervisors			Residents			Nurses	
	*M* ± *SD*	*N*	*p* ^*a*^	*M* ± *SD*	*N*	*p* ^*a*^	*M* ± *SD*	*N*
Responsibility	3.61 ± 0.86	54	<.001	3.72 ± 0.98	54	<.001	4.37 ± 0.76	67
Teamwork and collegiality	3.65 ± 0.69	57	<.001	3.82 ± 0.79	61	.012	4.19 ± 0.70	67
Knowing and maintaining own personal bounds and possibilities	3.39 ± 1.00	67	<.001	3.47 ± 0.78	58	.001	4.05 ± 0.77	63
Structure, work planning and priorities	3.22 ± 1.10	67	.001	3.36 ± 1.12	66	.016	3.87 ± 0.83	67
Coping with mistakes	3.62 ± 0.81	50	.003	3.63 ± 0.70	57	.003	4.14 ± 0.59	36
Scientifically and empirically grounded method of working	3.38 ± 0.91	48	<.001	3.38 ± 0.82	34	<.001	4.27 ± 0.63	22
Verbal communication with colleagues and supervisors	3.55 ± 1.00	67	<.001	3.65 ± 1.00	66	<.001	4.31 ± 0.72	67

^a^Significant differences compared with nurses

Participants received the highest ratings from physicians (supervisors and residents) for “teamwork and collegiality”, closely followed by “responsibility”. Nurses rated participants’ “responsibility” the highest. “Structure, work planning and priorities” received the lowest ratings by all three rating groups. “Coping with mistakes” as well as “scientifically and empirically grounded method of working” were the facet of competence that most frequently received the rating “judgement was not possible” by all groups of raters, documented by the lower numbers of ratings for these facets of competence in Table 1.

Nurses felt rather confident (all ratings on average > 3.7) in their judgement of all facets of competence (Table 2). Significant differences between nurses’ and supervisors’ confidence of judgment were found for the facets of competence “responsibility”, “teamwork and collegiality”, “coping with mistakes”, “scientifically and empirically grounded method of working”, and “verbal communication with colleagues and supervisors”. Supervisors felt least confident in assessing participants’ “coping with mistakes” and “scientifically and empirically grounded method of working” while residents felt least confident in assessing participants’ “responsibility”.

**Table 2:** Confidences of judgment by different rating groups

Competences	Supervisors			Residents			Nurses	
	*M* ± *SD*	*N*	*p* ^*a*^	*M* ± *SD*	*N*	*p* ^*a*^	*M* ± *SD*	*N*
Responsibility	3.47 ± 0.80	55	<.001	3.05 ± 0.81	66	<.001	4.23 ± 0.76	66
Teamwork and collegiality	3.04 ± 1.21	57	<.001	3.23 ± 0.97	64	<.001	4.25 ± 0.75	65
Knowing and maintaining own personal bounds and possibilities	3.64 ± 0.90	67	.901	316 ± 0.93	64	1.000	3.74 ± 1.02	65
Structure, work planning and priorities	4.04 ± 0.59	67	.256	3.73 ± 0.76	66	1.000	3.76 ± 0.96	67
Coping with mistakes	2.77 ± 1.25	52	<.001	3.46 ± 0.94	61	<.001	4.16 ± 0.81	43
Scientifically and empirically grounded method of working	2.47 ± 1.03	51	<.001	3.49 ± 0.94	41	<.001	4.23 ± 1.11	39
Verbal communication with colleagues and supervisors	4.09 ± 0.67	67	.209	3.58 ± 0.88	66	<.001	4.31 ± 0.70	67

^a^Significant differences compared with nurses

Facets of competence assessed by nurses show strong positive correlations with their confidence of judgment (Table 3). Supervisors’ assessments correlate only moderately with their confidence of judgment in two facets of competence: “verbal communication with colleagues and supervisors” with a positive and “scientifically and empirically grounded method of working”, with a negative correlation. Residents’ confidence of judgement correlates moderately with “teamwork and collegiality” and “knowing and maintaining own personal bounds and possibilities”.

**Table 3:** Correlations of assessed competences and confidence of judgment by rating groups

Competences	Supervisors		Residents		Nurses	
	*r* ^a^	*p*	*r* ^a^	*p*	*r* ^a^	*p*
Responsibility	.022	.873	.208	.125	.559	.000
Teamwork and collegiality	.216	.097	.435	.000	.581	.000
Knowing and maintaining own personal bounds and possibilities	.232	.054	.328	.011	.708	.000
Structure, work planning and priorities	.081	.507	.156	.199	.557	.000
Coping with mistakes	.021	.884	.164	.211	.365	.031
Scientifically and empirically grounded method of working	−.393	.004	−.006	.971	.655	.002
Verbal communication with colleagues and supervisors	.308	.009	.185	.128	.583	.000

^a^Including all ratings except cases in which judgement was not possible

## Discussion

According to the first aim of our study of comparing the three different assessor groups with respect to their rating scores for the different facets of competence, we found significantly higher ratings for all seven facets of competence by nurses compared to supervisors and residents, who both experienced the participants during the handover, which is probably the most relevant source for judgement. Another study, which assessed professionalism as well as interpersonal and communication skills in residents also showed that peers and consultants provided lower ratings than nurses [28]. The authors hypothesize that supervisors are probably more sensitised for professionalism, communication and interpersonal skills, which might have led to higher expectations by supervisors [28]. Since clinical or teaching experiences have an influence on assessors’ ratings [29], our supervisors’ ratings might be more critical than those of the nurses in our study, because of their clinical and teaching experience with a physician’s perspective. Additionally, the average agreement of 42.8% of both rating groups after adjusting for mean differences between nurses’ and supervisors’ scores might reflect different professional perspectives in different experienced situations within the assessment leading to different or similar scores for the facets of competence depending on participants’ performance. Since it is known from OSCEs that interpersonal skills are evaluated less reliably across stations [13] this difference might also be due to the fact that interpersonal skills or competences might be expressed differently in different situations and that the rater’s perspective is important to underscore the individual scoring with a personal reason and feedback to the participant. Supervisors and residents have similar professional perspectives, which might be another reason for similar ratings of these two rating groups in our study. The also observed the same interaction (patient handover), which would underscore the finding for OSCEs that rating of interpersonal skills was more reliable within stations [13]. Agreement with respect to the different facets of competence assessed in the study by Jani et al. was good between all rating groups [28]. In our study, all rating groups agree about “responsibilty” as one of the highest and “structure, work planning and priorities” as the lowest observed facets of competence. Even though the distribution of power between nurses and physicians was found to be asymmetric in favour of the physicians [30], both staff groups have certain responsibilities in patient care, while physicians bear the overall responsibility for the patient [31]. The highest assessment by supervisors and residents for “teamwork and collegiality” might be triggered by seeing their future colleagues in the medical students in the resident’s role. Hence, they might focus more on teamwork and collegiality while nurses have other demands with respect to physician-nurse collaboration [32] and other requirements of communication within this collaboration [30]. From the perspective of all three rating groups, participants received the lowest ratings for “structure, work planning and priorities”. In an analysis of the strain our participants perceived during the assessment, the highest strain level was found during the patient management phase [33]. This might reflect the lack of management competence observed by all raters.

The key finding of the second aim of our study, to analyze the confidence of rating judgments as an aspect of rater cognition, was, that the supervisors in our study gave more differentiated estimations of their confidence in their assessment decisions than the nurses and the residents, potentially triggered by supervisors’ greater experience in assessing competences [29] and by their generally higher expectations [28]. The positive correlations between assessed facets of competence and confidence of judgment in our study show, according to three-phase rater cognition models [3], that clear observability of a competence helps to give meaning to the expressed competence before forming a confident judgment. A highly developed facet of competence might be more visible, which could lead to higher confidence in the assessment. This seems to be more important for nurses, who are not as familiar as supervisors with assessing medical students and for whom more correlations were found between assessed facets of competence and confidence of judgement. A correlation between assessed facets of competence and confidence of judgement does not imply a better or worse quality of rating, but provides information with respect to the rating process itself. For supervisors, only two significant correlations of assessed facets of competence with the confidence of rating were found: a positive correlation for “verbal communication with colleagues and supervisors” and a negative correlation for “scientifically and empirically grounded method of working”. Therefore, for most factes of competence, other factors seem more relevant for supervisors to come to a judgement and feel confident with it. Since “scientifically and empirically grounded method of working” received the lowest rating of supervisors’ confidence and frequently could not be assessed at all, supervisors might have been afraid to make a wrong decision when assessing this facet probably due to a lack of observability [3].

A limitation of our study is, that the raters seem to lack possibilities to observe the participants’ competences for “coping with mistakes” and “scientifically and empirically grounded method of working”, because “no judgement possible” was marked most frequently for these two facets of competence. Furthermore, our rating form did not provide the opportunity to give a reason for the assessment. This hampers the assessment potential of our competence-rating tool.

A strength of our competence-rating tool, on the other hand, is the differentiated rating we received by the three different rating groups with no ceiling effect. Despite of the low sample size, we were able to identify significant differences between rating groups and significant correlations between assessment scores and confidence of judgment, as well as high effect sizes. The assessment format itself provides plenty of opportunities to develop and include scenarios, which make the facets of competence rated with less confidence more observable. Furthermore, a checklist with observable behavior as anchors for the different facets of competence, similar to the Group Assessment Performance (GAP)-test [34], will be developed and used for the rater trainings. Additionally, the rating form will be amended with a field to provide a reason for the rating of each facet of competence to reveal operationalizable factors for judgement related to the confidence of judgement. This support the rater’s perspective on a participant’s performance and provide arguments for individual feedback.

## Conclusions

The different professional perspectives provide differentiated competence ratings for medical students in the role of a beginning resident in interprofessional interactions. Since no significant differences were found between supervisors and residents, one rater per profession seems to be sufficient. To further decrease subjectivity and enhance confidence in rating competences with this assessment tool, empirically derived behavior checklists need to be developed for each facet of competence and need to be included in observational rater trainings.

## Additional file

Additional file 1: Sample of the rating form. (DOCX 30 kb)

## Abbreviations

CanMEDS: Royal College of Physicians and Surgeons of Canada
DOPS: Direct observed procedural skills assessment
Mini-CEX: mini-clinical evaluation
