# Supplementary material for: Interprofessional assessment of medical students’ competences with an instrument suitable for physicians and nurses
Source: BMC Med Educ. 2019 Feb 6;19:46. doi: 10.1186/s12909-019-1473-6 (PMC6364398; doi:10.1186/s12909-019-1473-6)
Supplement: Supplementary file 1 — Sample of the rating form. (DOCX 30 kb) [file 12909_2019_1473_MOESM1_ESM.docx]

| **Total judgement** | insufficient | | aceptable | | | very good | | no judgment possible |  | **Safety of judgement** | | | | | |
| --- | --- | --- | --- | --- | --- | --- | --- | --- | --- | --- | --- | --- | --- | --- | --- |
|  |  |  |  |  |  |  |  |  |  | uncertain | | | certain | | |
| **(1) Responsibility**  The physician takes responsibility and shows accountability for his work. He/She accepts liability for his work. | **O** | **O** | | **O** | **O** | | **O** | **O** |  | **O** | **O** | **O** | | **O** | **O** |
| **(2) Teamwork and collegiality**  The physician cooperates effectively and respectfully in a (multidisciplinary) team, taking the views, knowledge, and expertise of others into account. | **O** | **O** | | **O** | **O** | | **O** | **O** |  | **O** | **O** | **O** | | **O** | **O** |
| **(3) Knowing and maintaining own personal bounds and possibilities**  The physician knows the boundaries of his own ability and asks for help (timely) when needed. He/She reflects on himself and the situation. | **O** | **O** | | **O** | **O** | | **O** | **O** |  | **O** | **O** | **O** | | **O** | **O** |
| **(5) Structure, work planning and priorities**  The physician sees the overall picture, has organizational skills and a flexible attitude, and sets priorities in his work. | **O** | **O** | | **O** | **O** | | **O** | **O** |  | **O** | **O** | **O** | | **O** | **O** |
| **(6) Coping with mistakes**  The physician is aware of the fact that anyone can make and does make mistakes once in a while. He/She is approachable when someone points out his mistakes and reacts adequately when he thinks that a colleague makes a mistake. | **O** | **O** | | **O** | **O** | | **O** | **O** |  | **O** | **O** | **O** | | **O** | **O** |
| **(8) Scientifically and empirically grounded method of working**  The physician uses evidence-based procedures whenever possible and relies on scientific knowledge. He/She searches actively and purposefully for evidence and consults high-quality resources. He/She uses his scientific knowledge critically and carefully in his work. | **O** | **O** | | **O** | **O** | | **O** | **O** |  | **O** | **O** | **O** | | **O** | **O** |
| **(10) Verbal communication with colleagues and supervisors**  The physician gives structured, pithy, and unambiguous verbal reports on his/her findings on a patient and his diagnostic and therapeutic policy. He/She asks relevant and purposeful questions. | **O** | **O** | | **O** | **O** | | **O** | **O** |  | **O** | **O** | **O** | | **O** | **O** |
